# Neurodevelopmental disorder and juvenile-onset tics associated with microdeletion of the SRRM2 gene

**DOI:** 10.1007/s10072-025-08318-0

**Published:** 2025-06-23

**Authors:** Andrea E. Cavanna, Virginia Caimi, Elisa Capriolo, Gabriele Arienti, Anna Riva, Renata Nacinovich, Stefano Seri

**Affiliations:** 1 Department of Neuropsychiatry, Birmingham and Solihull Mental Health NHS Foundation Trust, National Centre for Mental Health, 25 Vincent Drive, Birmingham, B15 2FG UK; 2 Birmingham Medical School, School of Medical Sciences, College of Medicine and Health, University of Birmingham, Birmingham, UK; 3 College of Health and Life Sciences, Institute of Health and Neurodevelopment, Aston University, Birmingham, UK; 4 Child Neuropsychiatry, Fondazione IRCCS San Gerardo dei Tintori, Monza, Italy; 5 School of Medicine and Surgery, University of Milano-Bicocca, Milan, Italy

**Keywords:** SRRM2 gene microdeletion, Neurodevelopmental disorder, Juvenile-onset tics, Intellectual disability

## Abstract

**Background:**

SRRM2-related neurodevelopmental disorder is a recently described genetic diagnosis caused by loss-of-function variants. The clinical presentation is characterised by a developmental delay with mild intellectual disability, occasionally associated with features of autism spectrum disorder and/or attention-deficit/hyperactivity disorder, as well as inconsistent dysmorphic features, hypotonia, and obesity.

**Case description:**

We document the rare case of a 30-year-old man diagnosed with neurodevelopmental disorder and juvenile-onset tics associated with a microdeletion involving the SRRM2 gene. He initially presented with simple motor and vocal tics in early adulthood and subsequently developed handwriting tics and limb posturing (catatonic tics). Tic severity was rated as moderate-to-marked (Yale Global Tic Severity Scale score of 55/100) and treatment recommendations included alpha-2 agonists.

**Discussion:**

To date, a total of 37 cases presenting with loss-of-function mutations in SRRM2 have been reported as neurodevelopmental disease-causing mutations. Of these, 21 were males and none had tics as part of their neurodevelopmental manifestations. Our case report widens the spectrum of neurodevelopmental disorders observed in the context of SRRM2 gene microdeletions and prompts further research to disentangle the contributions of genetic and environmental factors to variable phenotypic expressions.

## Introduction

SRRM2-related neurodevelopmental disorder is a recently described genetic diagnosis caused by loss-of-function variants [1]. The SRRM2 gene is located on chromosome 16p and encodes the SRm300 protein, a splicing factor of the SR-related protein family. The serine- and arginine-enriched domains of SRm300 are involved in splicing regulation by promoting interactions between mRNA and the spliceosome catalytic machinery. SRRM2 also plays an important role in cellular and developmental processes during embryogenesis, emphasising the potential relevance of SRRM2 mutations to neurodevelopmental phenotypes [2]. In 2020, a landmark report described the clinical phenotypes of 22 patients with loss-of-function variants in SRRM2, including two patients with microdeletions [3]. This gene, predicted to be intolerant to loss-of-function and highly conserved through evolution, had not been previously reported in constitutive human disease.

The clinical presentation of SRRM2-related neurodevelopmental disorder is characterised by a mild developmental delay, occasionally associated with features of autism spectrum disorder (ASD) and/or attention-deficit/hyperactivity disorder (ADHD). Intellectual disability disorder (IDD) tends to be mild, whereas facial dysmorphism, hypotonia, and obesity and have been inconsistently reported. Here, we document the rare case of a patient with neurodevelopmental disorder who presented with juvenile-onset tics associated with microdeletion of the SRRM2 gene.

### Case report

A.B., a 30-year-old man with mild IDD, was referred to the specialist Tourette syndrome Clinic, Department of Neuropsychiatry, BSMHFT and University of Birmingham (United Kingdom), for the assessment and management of multiple motor and vocal tics. Delivery was reported as normal, whereas early neurodevelopmental milestones were delayed (not sitting independently by 12 months, not walking by 18 months, not using two-word phrases by 24 months). Following an initial diagnosis of global developmental delay, A.B. developed mild learning difficulties that prompted admission to a special school. Despite his neurodevelopmental symptoms, A.B. achieved reasonable social skills and a satisfactory level of functioning by early adulthood.

At the age of 25, while medication-free, A.B. developed repetitive movements that became progressively more complex and severe over time, ranging from facial twitching, ocular movements, mouth pulling, neck jerking, and limb posturing. A.B. also reported repetitive vocalisations, mainly in the form of clicking sounds. Neurological examination was unremarkable. The intermittent motor and vocal manifestations observed throughout the consultation and on home video recordings were consistent with juvenile-onset motor and vocal tics. A.B.’s tics were characteristically preceded by premonitory urges and were described as temporarily suppressible at the expense of mounting inner tension. A.B. reported spontaneous fluctuations in both tic frequency and severity (waxing and waning course). Anxiety was reported as his main tic-exacerbating factor, whereas active concentration was associated with temporary improvements. There were no pali/echo/coprophenomena or other socially inappropriate behaviours. A.B.’s speech and written production progressively deteriorated over time, concomitant to worsening speech arrest and handwriting tics. At the time of evaluation, his Yale Global Tic Severity Scale score was 55/100, indicating moderate-to-marked severity.

Both neuroimaging (magnetic resonance imaging) and electroencephalography were reported as unremarkable. There was no family history of tics. Clinical genetics (microarray analysis) revealed a pathogenic heterozygous microdeletion at 16p13.3 including the SRRM2 gene. This deletion was classified as pathogenic and associated with key features of the observed clinical phenotype. Over the preceding five years, A.B. had been prescribed serotonergic and antidopaminergic pharmacotherapy, which he had discontinued because of perceived lack of beneficial effects in the longer term. Recommendations were made for a first-line anti-tic agent (alpha-2 agonist Clonidine) for prolonged relapses in tic severity.

## Discussion

To the best of our knowledge, a total of 37 cases of loss-of-function mutations in SRRM2 have been reported as disease-causing mutations in previous studies [3–5] and case reports [2, 6, 7] of neurodevelopmental disorders. Cuinat et al. first established SRRM2 as a gene responsible for a rare neurodevelopmental disease in 22 subjects (14 males, mean age 11 years, range 4–28 years) presenting with developmental delay, facial dysmorphism (20/22), mild IDD (16/20), ASD (9/22), hypotonia (9/22), obesity (7/22), and ADHD (6/22) [3]. A related publication by Sheng et al. reported an 8-year-old girl with a deletion affecting SRRM2 along with 16 additional genes, associated with developmental delay, IDD and ADHD [6]. Pagnamenta et al. identified six unrelated patients (3 males and 3 females, age range 5–16 years) from the 100 K Genomes Project with loss-of-function variants in SRRM2 associated with IDD and facial dysmorphism, as well as ADHD (5/6), ASD (4/6), hypotonia (4/6), and obesity (2/6) [4]. Regan-Fendt et al. reported three new patients with SRRM2 loss-of-function pathogenic variants out of approximately 3100 clinical exome sequencing procedures performed at a single tertiary children’s hospital [5]. These patients– a 3-year-old male, a 24-year-old male, and a 17-year-old female– presented with developmental delay, IDD, ADHD, and facial dysmorphism, inconsistently associated with hypotonia (2/3), ASD (1/3), and obesity (1/3). Zhang et al. described a family (a pregnant woman of unspecified age plus her mother, brother, and daughter) with a nonsense mutation in the SRRM2 gene associated with delayed language development, IDD, and facial dysmorphism [7]. Finally, Chang et al. identified a novel pathogenic SRRM2 mutation in a 17-year-old male with speech delay, IDD, hypotonia, dysmorphism, and obesity [2].

We reported a further male subject diagnosed with developmental delay and mild IDD, without evidence of hypotonia, dysmorphic features, or obesity. Of note, as part of his neurodevelopmental symptoms he reported simple motor and vocal tics, as well as more complex catatonic tics [8] and handwriting tics [9]. The development of tics in early adulthood is atypical, as the average age at onset of neurodevelopmental tics is around 6 years. Rare cases of late-onset tic disorders (“tic disorder not otherwise specified”) have been documented [10]. From the limited data available, adult-onset tics appear to share some but not all features with childhood tics, as they occur predominantly in males, are often both motor and vocal in the same individual, tend to wax and wane, and are characterised by a poor response to treatment. Tic disorders, including Gilles de la Tourette syndrome, are genetically heterogeneous conditions, with contributions from multiple genes and environmental factors, which contribute to phenotypic variability [11, 12]. Taken together with previous reports, our findings highlight the potential role of the SRRM2 gene in the pathogenesis of selected phenotypes of both tic disorders and more common tic-related conditions, including ADHD and ASD.

In conclusion, we have presented a rare case of SRRM2 gene microdeletion associated with juvenile-onset tics, widening the spectrum of neurodevelopmental disorders observed in the context of SRRM2 gene microdeletions. While evidence supports the involvement of SRRM2 in the pathogenesis of neurodevelopmental phenotypes, further research is needed to clarify its specific role and contribution relative to other genetic and environmental factors.
